# Periodic F-actin structures shape the neck of dendritic spines

**DOI:** 10.1038/srep37136

**Published:** 2016-11-14

**Authors:** Julia Bär, Oliver Kobler, Bas van Bommel, Marina Mikhaylova

**Affiliations:** 1 DFG Emmy Noether Group ‘Neuronal Protein Transport’, Center for Molecular Neurobiology, ZMNH, University Medical Center Hamburg-Eppendorf, 20251 Hamburg, Germany; 2 Combinatorial Neuroimaging Core Facility (CNI), Leibniz Institute for Neurobiology, 39118 Magdeburg, Germany

## Abstract

Most of the excitatory synapses on principal neurons of the forebrain are located on specialized structures called dendritic spines. Their morphology, comprising a spine head connected to the dendritic branch via a thin neck, provides biochemical and electrical compartmentalization during signal transmission. Spine shape is defined and tightly controlled by the organization of the actin cytoskeleton. Alterations in synaptic strength correlate with changes in the morphological appearance of the spine head and neck. Therefore, it is important to get a better understanding of the nanoscale organization of the actin cytoskeleton in dendritic spines. A periodic organization of the actin/spectrin lattice was recently discovered in axons and a small fraction of dendrites using super-resolution microscopy. Here we use a small probe phalloidin-Atto647N, to label F-actin in mature hippocampal primary neurons and in living hippocampal slices. STED nanoscopy reveals that in contrast to β-II spectrin antibody labelling, phalloidin-Atto647N stains periodic actin structures in all dendrites and the neck of nearly all dendritic spines, including filopodia-like spines. These findings extend the current view on F-actin organization in dendritic spines and may provide new avenues for understanding the structural changes in the spine neck during induction of synaptic plasticity, active organelle transport or tethering.

Synapses form the basis for neuronal communication and the storage of information in the brain. The majority of excitatory synapses are found on dendritic spines. Their three-dimensional organization is believed to restrict the spread of ions and biochemical signals into the dendrite and to compartmentalize synaptic proteins and signalling molecules^1^,^2^. Electron and fluorescence microscopy data have shown that dendritic spines can be roughly divided into thin filopodia-like spines, short stubby spines without a clearly distinguishable neck, and mushroom-like spines consisting of a bulb-like head connected to the dendritic shaft via a thin extended spine neck^3^,^4^. The latter type is the most predominant in adult neurons^5^. Spine necks can be up to a few micrometers long and have a median width of about 150 nm^1^. Interestingly, synaptic potentiation induces a widening and shortening of the spine neck^1^. In addition, spines are very plastic. They continuously change their shape and size or become stable over certain periods of time and can appear and disappear in response to synaptic activity^6^. Such dynamics are mediated by rapid changes in the actin cytoskeleton^7^.

It is therefore important to gain better understanding of the nanoscale organization of the actin cytoskeleton in dendritic spines. Conventional fluorescence microscopy in combination with photoactivatable probes or fluorescence recovery after photo-bleaching (FRAP) is frequently used to study protein localization or protein dynamics in spines^8^,^9^. However, due to the diffraction of light, it is not possible to resolve structures below approximately half of the wavelength used to excite the fluorophore, which is about 250 nm. Electron microscopy (EM) has provided deeper insights into the nanoscale organization of dendritic spines^10^. Although, a major drawback of this method is that it is difficult to visualize F-actin by conventional preparation of samples^11^,^12^. Replica EM has been successfully used to study actin filaments in primary hippocampal neurons^13^ and these findings contribute to the current model of F-actin organization in spines, in which the spine head contains a dense network of branched actin filaments, which decrease in density towards the head-neck junction. Structural support of the spine neck is mediated by branched and linear longitudinal actin filaments loosely cross-linked by myosin II. The base of the spine contains linear and branched filaments with the first type being more predominant^13^.

In the past years different super-resolution microscopy techniques have emerged that allow diffraction-unlimited imaging^14^. For instance, single molecule localization microscopy techniques (SMLM: i.e. STochastic Optical Reconstruction Microscopy, STORM, etc.) and Stimulated Emission Depletion (STED) nanoscopy offer a resolution down to 10–50 nm and 35 nm, respectively, and allow imaging of fixed or living samples^15^,^16^,^17^,^18^. In combination with the highly specific protein labelling associated with multi-color fluorescence microscopy, these methods are well suited to study the properties of the dense neuronal cytoskeleton. Further significant steps, in improving the accuracy of localization for proteins, have been made with the development of novel small probes and fluorescent labels^16^,^19^,^20^,^21^.

One of the most significant discoveries made by super-resolution microscopy is the periodic organization of the subcortical actin and spectrin lattice found in axons and 10–30% of the dendrites^17^,^20^,^22^,^23^,^24^. These ring-like structures appear with 180–190 nm periodicity and have been found in both vertebrate and invertebrate species, in all types of neuronal cells as well as in precursors of oligodendrocytes^24^,^25^. A very recent study describes a similar periodic spectrin pattern in the neck region of approximately 25% of spines, visualized by STORM microscopy and anti β-II spectrin antibody labelling^24^. A parallel study reported that a β-II spectrin lattice is present in dendrites of adult hippocampal neurons and can continue into thicker spine necks, but its immunoreactivity is generally absent from the PSD^18^. Both reports found that direct identification of F-actin periodicity in spines by using phalloidin, a small fungal toxin, which binds actin filaments, was difficult. Taking into account that actin rings have not been detected by replica EM^13^ and that β-II spectrin may have additional functions, it is currently not clear whether actin filaments in spine necks are also arranged in periodic patterns.

We aimed to gain further insights into the structural organization of dendritic spines and established a protocol for labelling and detection of periodic actin structures in adult primary neurons and live hippocampal slices using 2-color STED nanoscopy with phalloidin-Atto647N and β-II spectrin labelled by secondary antibodies conjugated to Abberior Star 580. We found that phalloidin-Atto647N stains a periodic actin lattice in all dendrites and, in contrast to β-II spectrin, the neck of nearly all dendritic spines including the mushroom-like spines with a long thin neck and less mature filopodia-like spines. These finding modify the current model of F-actin organization in dendritic spines^26^ and may provide new avenues for understanding the structural changes in spine necks during induction of synaptic plasticity, active organelle transport or tethering.

## Results

### Periodic cortical F-actin cytoskeleton is a general feature of dendritic spine necks in primary hippocampal neurons

As an alternative to antibody labelling, the smaller compounds SiR-actin and phalloidin have been successfully applied to directly visualize F-actin in axons and dendrites of primary neurons using STED nanoscopy. SiR-actin is a bright far-red shifted dye well suited for live labelling of actin filaments^17^,^20^. However, upon 4% PFA fixation, which is required for antibody staining, the signal becomes much dimmer. Phalloidin-Atto532 was recently used for STED imaging of the actin/spectrin lattice in dendrites, but in contrast to dendritic shafts, periodic F-actin structures in spine necks were not observed^18^. For our studies we took advantage of depletion of two dyes, Abberior Star 580 and Atto647N, with one depletion laser (775 nm) to ensure perfect conditions for co-localization studies. We established a protocol that allows for triple fluorescence labelling: dendrites are visualized by an antibody directed against MAP2 coupled to AlexaFluor488 or by overexpressed GFP as a cell fill, spectrin lattice or synapses by antibodies against β-II spectrin or the synaptic marker bassoon labelled with Aberrior Star 580, and actin filaments by phalloidin conjugated to Atto647N (A647N), a far-red high quantum yield fluorophore for STED imaging^27^. In a series of tests we found that following immunostaining of freshly fixed adult hippocampal primary neurons an overnight incubation with 0.165 nM phalloidin-A647N results in a strong A647N fluorescence signal in dendrites and dendritic spines (Supplementary Fig. S1). STED imaging of dendrites detected by MAP2 fluorescence in the confocal mode revealed that all dendrites, independently of the distance from the soma, contained 3 types of actin filaments: longitudinal actin fibres, actin patches and periodically organized cortical actin Supplementary Fig. S1, I-III).

**Figure 1:**
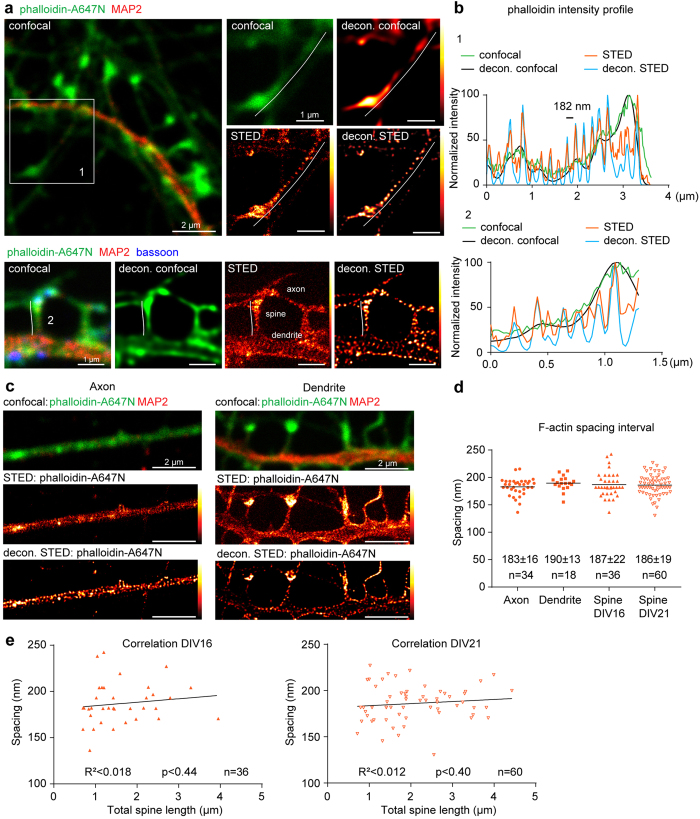
Periodic F-actin lattice is present in nearly all necks of dendritic spines. (a) Top: Confocal image of a primary hippocampal neuron (DIV21) stained with anti-MAP2 antibodies and phalloidin-A647N with corresponding raw/deconvolved confocal and STED images of F-actin in higher magnification. Bottom: Confocal image of a mushroom-like spine from primary hippocampal cultures stained for actin (phalloidin), MAP2 and the presynaptic marker bassoon with corresponding deconvolved confocal, STED and raw STED image for phalloidin. Lines for intensity profile measurement (**b**) are indicated. (**b**) Normalized line profiles of phalloidin-Atto647N intensity along the spine necks indicated in (**a**) for raw and deconvolved confocal and STED images. (**c**) Representative confocal, raw and deconvolved STED images of periodic actin structures in axon (left) and dendrite (right). (**d**) Quantification of cortical F-actin periodicity in axons, dendrites and spines at DIV16 and DIV21 shows no difference in spacing interval. Indicated are mean ± standard deviation (Brown-Forsythe test for equal variances p = 0.20, 1-Way-ANOVA p = 62). Numbers of analyzed spines (n), dendritic or axonal segments (maximum 2 per neurite). from 3 independent experiments. (**e**) Periodic actin spacing does not correlate with spine length. The number of analyzed spines (n), Linear correlation (black line), coefficient of determination (R^2^) and p value (for slope being non-zero) are indicated.

Interestingly, a periodic pattern in phalloidin-A647N labelling could be observed in the neck of dendritic protrusions of different length and morphology (Fig. 1a). To confirm that the dendritic protrusions were functional synapses containing a pre-synaptic part, we performed 2-color STED imaging using the pre-synaptic marker bassoon (Fig. 1a, lower panel). To further improve the images we deconvolved the original acquisitions with Huygens Professional software (Fig. 1a; also see Methods). The line intensity profile along the dendritic protrusions revealed a similar periodicity of actin rings as it was observed in axons and dendrites in STED and deconvolved STED channels but not in the deconvolved confocal images (Fig. 1b,c, Supplementary Fig. S2a). Quantitative analysis performed on the original STED acquisitions showed that the F-actin lattice had an average peak interval of 187 ± 22 nm (n = 36 spines) in spine necks at DIV16, and 186 ± 19 (n = 60 spines) at DIV21, 183 ± 16 nm (n = 34 axon stretches) in axons, and 190 ± 13 nm (n = 18 dendritic stretches) in dendrites (Fig. 1d). Shorter stretches of periodic F-actin in dendrites, as also partially observed here, are indicative for the looser organization of cortical actin cytoskeleton in dendrites^24^ (Fig. 1c). The periodicity interval of F-actin in spine necks did not significantly differ from those in the axon or the dendrites and did not vary between the neurons of DIV 16 or DIV 21 (1-way ANOVA p = 0.62, Fig. 1d). A Fast Fourier Transform (FFT) algorithm applied to an individual long spine (3 μm spine neck with at least 12 rings) revealed a peak corresponding to a 189 nm periodicity with a similar strength of periodic nature between the raw and the deconvolved STED images (Supplementary Fig. S2b).

**Figure 2:**
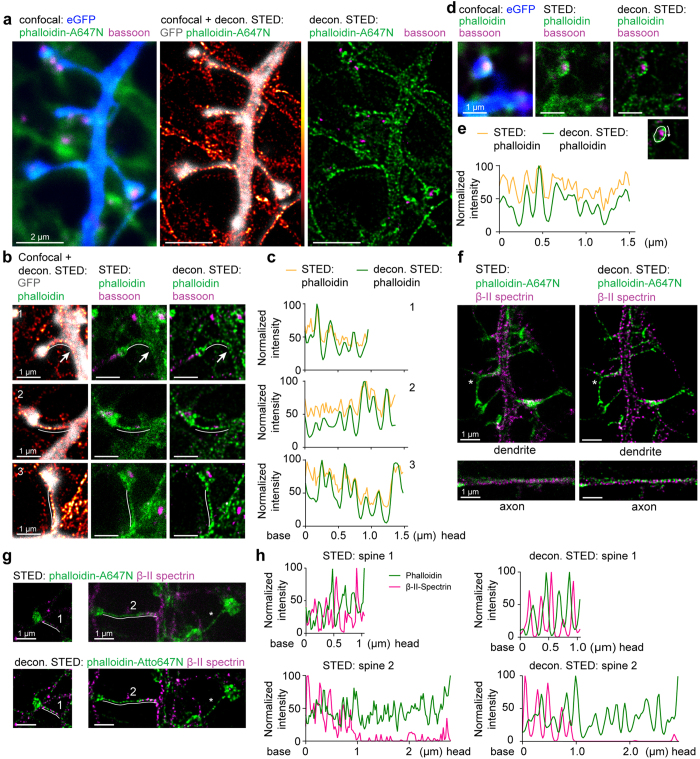
Periodic structures are found in mushroom-like spines of primary hippocampal neurons and can be better identified by phalloidin-A647N than β-II spectrin staining. (**a**) Confocal image of a DIV16 hippocampal neuron filled with eGFP (blue) and stained for phalloidin (green) and bassoon (magenta) with corresponding deconvolved STED images. Note that phalloidin fills the complete dendrite and spines, and that periodic actin structures are found in all spines. (**b**) Periodic actin structures in mushroom-like spines of hippocampal primary cultures (DIV16) filled with eGFP and stained for phalloidin and bassoon (left), including corresponding raw and deconvolved STED images for phalloidin and bassoon. (**c**) Normalized intensity profiles of spines indicated in (**b**). (**d**) Periodic actin patterns can extend into the spine head. Confocal image of an eGFP-filled spine stained for phalloidin and bassoon at the presynaptic site, and corresponding raw and deconvoled STED image of phalloidin and bassoon. Note the localization of bassoon in the center of the phalloidin staining. (**e**) Normalized intensity profile of actin structures in the spine head shown in (**d**) along the line indicated in the inlet. (**f**) 2-Color raw and deconvolved STED images of β-II spectrin (magenta) and phalloidin-A647N (green) show a periodic F-actin/spectrin organization in axons and dendrites. *indicates spines with β-II spectrin entering a spine base. (**g**) Phalloidin-A647N (green) is better suited to visualize periodic cytoskeleton organization than β-II spectrin stainings (magenta). Spine 1 represents an example where the cortical cytoskeletal lattice is organized by alternating phalloidin-A647N and β-II spectrin labelling. In case of spine 2, β-II spectrin is present at the beginning of the spine neck but then disappears, whereas F-actin periodicity is observed throughout the entire neck. *Indicates a spine lacking β-II spectrin stainings completely. Corresponding confocal images and MAP2 staining are in the Fig. S3. (**h**) Normalized intensity profiles of raw and deconvolved STED indicated in (**g**).

Dendritic spines are highly dynamic structures. As they continuously move, grow and shrink it might be possible that the distance between periodic F-actin structures changes during such motion. We aimed to explore what is the relationship between the spine length, spine type and the periodic pattern of the F-actin staining. From 239 dendritic protrusions (DIV21) the total protrusion length was varied between 0.3 to 4.3 μm with the majority ranging from 0.8 to 2 μm (Figs 1a and 2a). Therefore we selected approximately equal numbers of representative spines of each length category (total 60 spines from DIV21 and 36 from DIV16 spines; 3 independent experiments) and performed a correlation analysis between the spatial period of F-actin labelling and the spine length (Fig. 1e). We found that the periodicity did not correlate with the length of the protrusion, neither was it influenced by the age of culture (R^2^ < 0.018 at DIV21, R^2^ < 0.012 at DIV16; Fig. 1e). In addition, dendritic protrusions categorised by type based on the presence of pre-synaptic terminals (spines vs. filopodia or lamellipodia) and the ratio between the width of the spine neck and the head (mushroom-like, long mushroom-like vs. thin spines) did not significantly differ in the frequency of periodic F-actin elements (1-way ANOVA p = 0.62, p = 0.41, Supplementary Fig. S3b, see Methods section for description of spine classification).

Next, we wondered how well the phalloidin labelling reflects spine morphology. Transfection of DIV16 neurons with a GFP fill allowed for a standardized morphology read out (Fig. 2a–e). The entire spine volume was filled with F-actin indicating that phalloidin staining can be used for assessment of the spine shape. Interestingly, in contrast to the previous finding where β-II spectrin periodic structures extended only to the base region of spines with thicker necks^18^, we detected the periodic F-actin lattice in spines of various shapes (Fig. 2a,b, Supplemental Fig. S3c,d). Frequently, the periodic pattern continued from the dendritic shaft into the spine neck, persisted throughout the entire length and even extended into the head of mushroom-like spines (Fig. 2d,e, Supplemental Fig. S3c–e; in total about 13 cases with periodic actin structures in the spine head were observed).

Next, we investigated the relationship between F-actin and β-II spectrin staining in dendritic spines. Alternating patterns of F-actin and spectrin, as reported for axons and dendrites (Fig. 2f, Supplemental Fig. S3f), were also found in a subset of spine necks^18^ (Fig. 2g). However, β-II spectrin immunoreactivity was not detected in every spine neck, (approximately 50% of spines displaying F-actin periodicity; Fig. 2g,h, Supplemental Fig. S2g), and often not along the complete length where the periodic phalloidin pattern is seen (Fig. 2g,h). Notably, in these spines phalloidin-A647N was still found in a periodic manner (Fig. 2g,h, Fig. S2g).

### Periodic F-actin structures are present in the neck of dendritic spines *in vivo*

**Figure 3:**
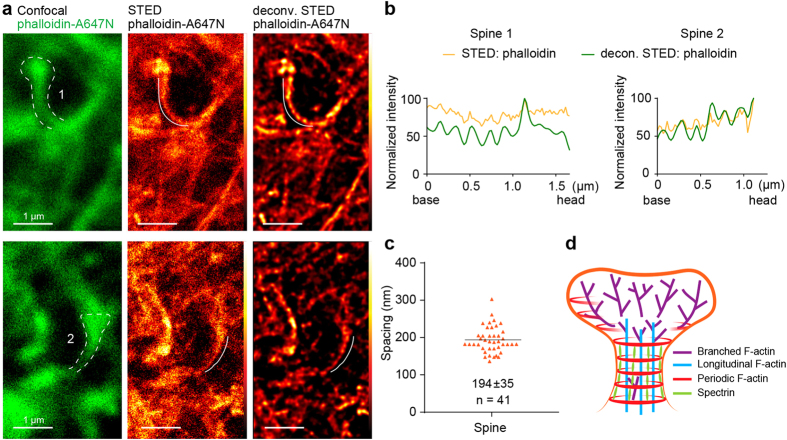
Phalloidin-A647N labels the periodic actin lattice in dendritic spines in acute hippocampal slices. (**a**) Representative confocal images of 2 spines from hippocampal slices with phalloidin-Atto647N (green, left panel) and corresponding raw and deconvolved STED image (middle/right). (**b**) Normalized intensity profiles of phalloidin-A647N of the spines indicated in (**a**). More examples are in the Fig. S4. (**c**) Quantification of the spacing of periodic actin structures in spine necks of hippocampal slices. Mean ± standard deviation is indicated. (n) Number of analysed spines from 5 slices in 2 independent experiments. (**d**) Model of the cytoskeleton organization in dendritic spines.

*In vivo* and slice culture studies have shown that the morphology of neurons in adherent primary neuronal culture can differ from their 3D organization in the brain. This is also true for synaptic connectivity and the representation of spine types^1^. Therefore, our next step was to verify if the periodic actin organization is also a feature of spine necks in tissue slices. Staining and imaging of actin in brain tissue is challenging due to the high density and tight spacing between the cells. We exploited the fact that living neurons, but not other cell types in brain, are permeable to phalloidin in a dose-dependent manner^28^. We initially tested the protocol in organotypic hippocampal slice cultures (Supplemental Fig. S4a,b; see Methods section). A small droplet of phalloidin-A647N (6.6 μM) was pipetted on top of the slice and incubated for >0.5 hour (Supplemental Fig. S4a). The probe entered neurons in the upper layer and spread along the dendritic tree deeper into the slices allowing selective labelling of dendrites and spines (Supplemental Fig. S4b). Imaging of those brain slices revealed the presence of periodic phalloidin staining in dendritic spines (Supplemental Fig. S4c,d). Of note, the use of far-red shifted dyes is beneficial for imaging in deeper tissue because of the better penetration of longer wavelengths and less autofluorescence. Next, for *in situ* labelling of F-actin, we prepared 400 μm thick acute hippocampal slices from 9-week old rats. After several hours of tissue recovery, we applied phalloidin-A647N as it was optimized for organotypic slices (Supplemental Fig. S4a). Even though it is more difficult to focus on the longitudinal axis of spines in 3D, we were able to identify periodic arrangement of F-actin in the spine neck and the dendrites (Fig. 3a–c, Supplemental Fig. S4e,f). The mean spacing between these structures in the spine neck was not different from the interval found in the primary hippocampal neurons (194 ± 35, n = 41 in slices and 186 ± 19, n = 60 in DIV21 primary neurons, Student’s t-test p = 0.16) suggesting that this type of cortical cytoskeleton organization is a general feature of dendritic spines.

## Discussion

In this work we used phalloidin-A647N to examine the nanoscale organization of actin filaments in dendritic spines. 2-color STED imaging allowed us to the study the relationship between F-actin and β-II spectrin, or bassoon as a presynaptic marker. Phalloidin conjugated to Atto647N turned out to be well-suited for STED nanoscopy of various actin structures. The small size of this probe has potential advantages compared to conventional antibodies and can improve the labelling of fine structures embedded in very dense environments^16^, like dendritic spines. Periodic actin rings with intervals around 190 nm could be resolved in all axons, dendrites, and nearly all neck regions of dendritic spines. Notably, in comparison to phalloidin-Atto647N, periodicity in β-II spectrin labelling was as well found in axons and dendrites but was less common in dendritic spines and often restricted to the spine base. This indicates that phalloidin may penetrate more effectively into densely packed cellular compartments than the spectrin antibody. It is also important to mention that a fraction of the spines may contain different spectrin isoforms contributing to the organization of cortical actin cytoskeleton^29^. These spines may differ, as a consequence, in their plastic properties. Moreover, the notion of irregular organization of cortical cytoskeleton in dendrites with isolated patches of periodic cytoskeletal structures is based on β-II spectrin stainings rather than direct actin labelling^24^. In our experiments phalloidin-Atto647N decorated a periodic actin lattice in all dendrites, which indicates a potential involvement of other spectrin isoforms different than a disruption of the periodic actin pattern. At last, live STED imaging with another actin probe, SiR-actin, could show the periodic F-actin lattice only in 10–30% of the dendrites^17^. This discrepancy may come from the fact that SiR-actin has a higher on/off binding rate to actin filaments than phalloidin and, as a consequence, it is possible that a higher actin turnover in some of the dendrites led to blurring of the STED image

We found that periodical arrangements of cortical actin fibers are a common feature of all spine types. They are present in the neck of mature mushroom-like spines with necks of various length and width, as well as in less mature filopodia-like spines. These periodic F-actin elements frequently begin at the membrane of the dendritic shaft and extend into the spine neck. Interestingly, we found no correlation between the periodicity of F-actin structures and the length and type of dendritic spines. This suggests that the flexibility of the spine neck is most likely mediated by rapid formation and disassembly of the acting rings rather than changes in the spacing interval. A possible explanation for the absence of a periodic actin lattice in EM images may be a difference in the fixation protocol. We performed fixation of primary neurons with 4% PFA and 4% sucrose in PBS buffer whereas for replica EM samples the plasma membrane needs to be pre-extracted first, exposing the cytoskeleton, before fixation^13^. It is possible that the cortical actin network, similarly to some PSD components, is either removed or partially destroyed by this procedure.

The presence of periodic F-actin structures within spine necks, both in primary culture and brain slices changes the current view of actin organization in spines. While the inner part of the spine neck consists of loosely aligned longitudinal and branched actin filaments with the ratio strongly shifting towards the branched ones in a spine head^13^, the plasma membrane of the spine neck is decorated with periodic actin filaments (Fig. 3d). Those actin rings could provide mechanical support for the spine neck, which is constantly shaped by synaptic activity. Future research will shed light on the 3-dimentional aspects of the cortical actin organization, for instance, how periodic F-actin in a spine neck relates to periodic F-actin found in a subset of spine heads; or whether periodic actin in analogy to axons consist of rings, and if these rings are closed or arranged in a spiral. This may directly affect the biophysical properties of the neck, including its flexibility and elasticity and its susceptibility towards activity induced changes. At the moment it remains unclear which other actin binding proteins are involved in the organization of F-actin within spines lacking β-II spectrin. Considering the high number of actin nucleation factors and modulators of actin dynamics contributing to the plastic changes occurring in dendritic spines, it is important to unravel their involvement in the formation of the actin/spectrin lattice. Another exciting question is how periodic actin structures are involved in spine neck elasticity during transport of large and bulky organelles in and out of spines and thereby contribute to synaptic plasticity.

## Methods

### Hippocampal neuronal primary cultures, transfections and immunocytochemistry

Sacrificing of animals was done in accordance with the Animal Welfare Law of the Federal Republic of Germany (Tierschutzgesetz der Bundesrepublik Deutschland, TierSchG) and with the approval of local authorities of the city-state Hamburg (Behörde für Gesundheit und Verbraucherschutz, Fachbereich Veterinärwesen, from 21.04.2015) and the animal care committee of the University Medical Center Hamburg-Eppendorf. For the procedure of sacrificing rats for subsequent isolation of hippocampal neurons or preparation of hippocampal slices, all regulations and guidelines given in §4 TierSchG are followed. According to §7 Abs. 2 Satz 3 TierSchG sacrificing of animals is not an experiment on animals, therefore no specific authorization or notification is required. Primary hippocampal cultures were prepared with slight modification as described previously^30^. Shortly, brains of E18 Wistar rat embryos were dissected, hippocampi removed, washed in ice cold Hanks’ balanced salt solution (HBSS, Sigma Aldrich), and trypsinized for 15 min by addition of 100 μl per hippocampus of 0.25% trypsin/0.02% ethylenediaminetetraacetic acid (EDTA, Thermo Fisher Scientific) at 37 °C. After washing with warm HBSS, cells were triturated by repeated pipetting through glass needles (20 G, 26 G), filtered via a 100 μm strainer (Greiner), counted and plated in 1 ml DMEM (Thermo Fisher Scientific supplemented with 10% foetal calf serum, 2 mM glutamine, and 1x penicillin/streptomycin) on poly-L-lysin coated glass coverslips at a density of 30000 cells per coverslip in 12-well-plates. The medium was exchanged to neurobasal medium (Thermo Fisher Scientific, supplemented with 2% B27, 0.25% glutamax, 1x penicillin/streptomycin) after cells adhered. Cells were incubated in an incubator at 37 °C, 5% CO_2_ and 95% humidity. At DIV 15 neurons were transfected with pEGFP-N1 (Clontech) as a cell fill using Lipofectamine2000 (Invitrogen) as described earlier^30^ and fixed with 4% PFA (Roti-Histofix, Carl-Roth)/4% sucrose in PBS for 10–15 min at room temperature (RT) at DIV16. Non-transfected cells were fixed at DIV21 in a same way as DIV16, washed with PBS and transferred into a humidified chamber for immunostaining. Cells were permeabilized for 10 min with 0.2% Triton X-100 in PBS, washed and blocked in blocking buffer (BB, 0.1% Triton X-100, 10% horse serum in PBS). Incubation with primary antibodies (mouse anti-β-II spectrin (BD Biosciences #612562, 1:150), mouse anti-bassoon (clone SAP7F407 Stressgen, 1:500) or mouse anti-cortactin (Millipore, 1:500), and rabbit anti-MAP2 (Abcam #ab32454, 1:400)) diluted in BB was done over night at 4 °C. Secondary antibodies (anti-rabbit-Alexa488 1:500, anti-mouse Abberior Star 580, Abberior GmbH Göttingen, Germany 1:200) were applied in BB for 1.5 h after 3 washing steps at RT. Incubation with phalloidin-Atto647N (0.165 nM in PBS) was performed at RT for 2.5 h and subsequently over night at 4 °C. Cells were washed with PBS and mounted onto objective slides with Mowiol (Sigma Aldrich).

### Preparation of organotypic and acute hippocampal slices and phalloidin uptake assay

Phalloidin staining in slices was optimized in organotypic hippocampal slice cultures prepared from P4-6 Wistar female rat pups. The hippocampi were dissected in ice-cooled dissection medium (in mM: 1 CaCl_2_, 5 MgCl_2_, 10 glucose, 4 KCl, 26 NaHCO_3_, 248 sucrose, 2 kynurenic acid, 0.001% phenol red, Sigma-Aldrich) and cut into 400 μm slices using a tissue chopper (McIlwain Mickle Laboratory Engineering, Surrey, UK). Slices were cultured on millicell membranes (Merck Millipore) in culturing medium (MEM (Invitrogen), containing 20% horse serum, 1 mM L-glutamine, 0.00125% ascorbic acid, 0.01 mg/ml insulin, 1 mM CaCl_2_, 2 mM MgSO_4_, 13 mM D-glucose, 1 M HEPES pH ~7,28, Sigma-Aldrich) and cultured in the incubator (5% CO_2_, 37 °C, and 95% humidity). Slices were fed twice per week by replacing 700 μl of the medium. Two to three weeks old organotypic slices cultures were stained with phalloidin-A647N by pipetting a small droplet (2–4 μl, 6.6 μM) on top of the slice. After >1 hour of incubation, the organotypic slices were imaged at an Olympus Fv1000 scanning Confocal/multi-photon upright microscope and the Abberior STED system.

Acute hippocampal slices were prepared from a 9–weeks old male Wistar rat as described above. Within 24 hours slices were live stained with phalloidin-Atto647N. A small droplet of Phalloidin-Atto647N (2–4 μl, 6.6 μM) was pipetted on top of the slice and imaged >1 hour after incubation at a Leica TCS SP8-3X gated STED microscope.

### Confocal and STED imaging

Overview confocal images of organotypic hippocampal slice cultures were acquired at an Olympus Fv1000 scanning confocal/multi-photon upright microscope controlled by Fluoview software (version 4.2). Slices were submerged in HEPES-buffered artificial cerebrospinal fluid (ACSF) containing (in mM): 145 NaCl, 10 HEPES, 12.5 D-glucose, 1.25 NaH_2_PO_4_, 2.5 KCl, 1 MgCl_2_, 2 CaCl_2_, (Sigma-Aldrich), pH ~7.4 at RT. Images were taken with an 25x water immersion objective (Olympus XLPlan N, 1.05 NA) with 3.5x zoom. Phalloidin-Atto647 was excited by a 635 nm laser and detected by an alkali-photodetector. Optimal labelling of dendritic structures and best signal-to-noise ratio was found ~10 μm below the slice surface.

Single plane STED images were acquired on a Leica TCS SP8-3X gated STED microscope equipped with a pulsed 775 nm depletion laser and a pulsed white light laser (WLL) for excitation. For acquiring images the Leica objective HC APO CS2 100x/1.40 oil was used.

Primary neuron samples were excited by the WLL at 650 nm (phalloidin), 488 nm (MAP2) and 561 nm (bassoon/β-II spectrin), respectively. MAP2 staining was only imaged in confocal mode. For STED imaging emission was acquired between 660–730 nm for Atto647N and 580–620 nm for Abberior Star 580. The detector time gates for both STED channels were set from 0.5–1 ns to 6 ns. Both dyes were depleted with 775 nm. Respective confocal channels uses the same settings as STED channels, except the excitation power was reduced and the detection time gates were set to 300 ps to 6 ns for both channels. The format for all images were set to 1024 × 1024 pixels. With an optical zoom of 5, the resulting voxel size is 23 nm for xy. Images were taken with 600 lines per second and line averaging of 16.

STED images from adult acute hippocampal slices were acquired by placing the slices up-side-down in a live imaging chamber contain HEPES-buffered ACSF (in mM: 145 NaCl, 10 HEPES, 12.5 D-glucose, 1.25 NaH_2_PO_4_, 2.5 KCl, 1 MgCl_2_, 2 CaCl_2_, (Sigma-Aldrich), pH ~7.4 at RT). The format for all images was set to 1024 × 1024 pixels. With an optical zoom of 6, the resulting resolution is 18.9 nm per pixel for x-y. Images where taken with 600 lines per second and line averaging of 8. Time gates were set to 0.5 to 6 ns.

STED images from organotypic slice cultures were acquired with Abberior Instruments STED microscope using 60x P-Apo oil objective 1.4 NA. The imaging conditions were the same as for acute slices. Phalloidin-A647N was excited by a 640 nm laser and depleted by a 775 nm pulsed laser. Pinhole size was set to 0.83 airy units, pixel size 20 nm.

### Data analysis

Spine length was measured manually in raw STED images in Fiji^31^ by drawing a segmented line from the spine base along the neck to the tip of the protrusion. Spines were defined as dendritic protrusions showing a clear presynaptic contact as visualized by bassoon or an enrichment of cortactin at the postsynaptic site, independent of spine length. Differentiation between mushroom and thin spines was purely defined by appearance of a clear spine head, which is at least 1.5x thicker than the neck. Mushroom-like spines were called ‘long mushroom’ when the total spine length was exceeding 2 μm. Protrusions without bassoon staining are classified as filopodia-like (same with over entire length) or lamelliopodia-like (amorphic protrusions). Data from at least three independent neuronal preparations for DIV21 and one for DIV16 were used for the analysis.

Intensity profiles of segmented lines (width1–3pixels) along axons, dendrites and spine necks were created in Fiji using the plot profile option, values exported and graphs generated in Prism 6 (GraphPad, La Jolla, CA, USA). Local maxima of intensities profiles of raw STED images were used for quantification of actin ring spacing. They were defined manually by a researcher blind to the identity of the measured profile. Inter-peak distances between at least 3 subsequent local intensity maxima were calculated.

To improve image quality for representation, raw data of STED images were deconvolved using the Huygens Professional (SVI, v 15.10) STED package as follows: to calculate the theoretical point spread function (PSF) the optical microscopic parameters provided by the lif-file itself were used. Within the Deconvolution wizard, images were subjected to a manual background correction. For deconvolution, the signal to noise ratio was set to 15. The Optimized iteration mode of the CMLE was applied until it reached a Quality threshold of 0.05.

For representation of confocal and STED images, contrast was enhanced by linear methods using Fiji for the complete image for each channel separately.

### Statistics

Differences in F-actin periodicity in spines, dendrites and axons was analysed by 1-way analysis of variances (ANOVA) after testing for equal variances between the groups (Brown-Forsythe test) using Prism 6. Correlation between spine length and actin spacing was analysed using linear regression mode in Prism 6. Prism 6 was also used to create all graphs. Origin 2015 (OriginLab Corporation) was used to perform fast Fourier transformation (FFT) of a single selected spine profile.

## Additional Information

**How to cite this article**: Bär, J. *et al*. Periodic F-actin structures shape the neck of dendritic spines. *Sci. Rep*. **6**, 37136; doi: 10.1038/srep37136 (2016).

**Publisher’s note**: Springer Nature remains neutral with regard to jurisdictional claims in published maps and institutional affiliations.

## Supplementary Material

Supplementary Information
