# Supplementary material for: Periodic F-actin structures shape the neck of dendritic spines
Source: Sci Rep. 2016 Nov 14;6:37136. doi: 10.1038/srep37136 (PMC5107894; doi:10.1038/srep37136)
Supplement: Supplementary Information [file srep37136-s1.doc]

**Supplementary Information**

**Periodic F-actin structures shape the neck of dendritic spines**

**Julia Bär**^1,+^**, Oliver Kobler**^2,+^**, Bas van Bommel**^1,+^**, and Marina Mikhaylova**^1^,*

^1^ DFG Emmy Noether Group 'Neuronal Protein Transport', Center for Molecular Neurobiology, ZMNH, University Medical Center Hamburg-Eppendorf, 20251 Hamburg, Germany

^2^ Combinatorial Neuroimaging Core Facility (CNI), Leibniz Institute for Neurobiology, 39118 Magdeburg, Germany

* corresponding author: marina.mikhaylova@zmnh.uni-hamburg.de

+ these authors contributed equally to this work

**
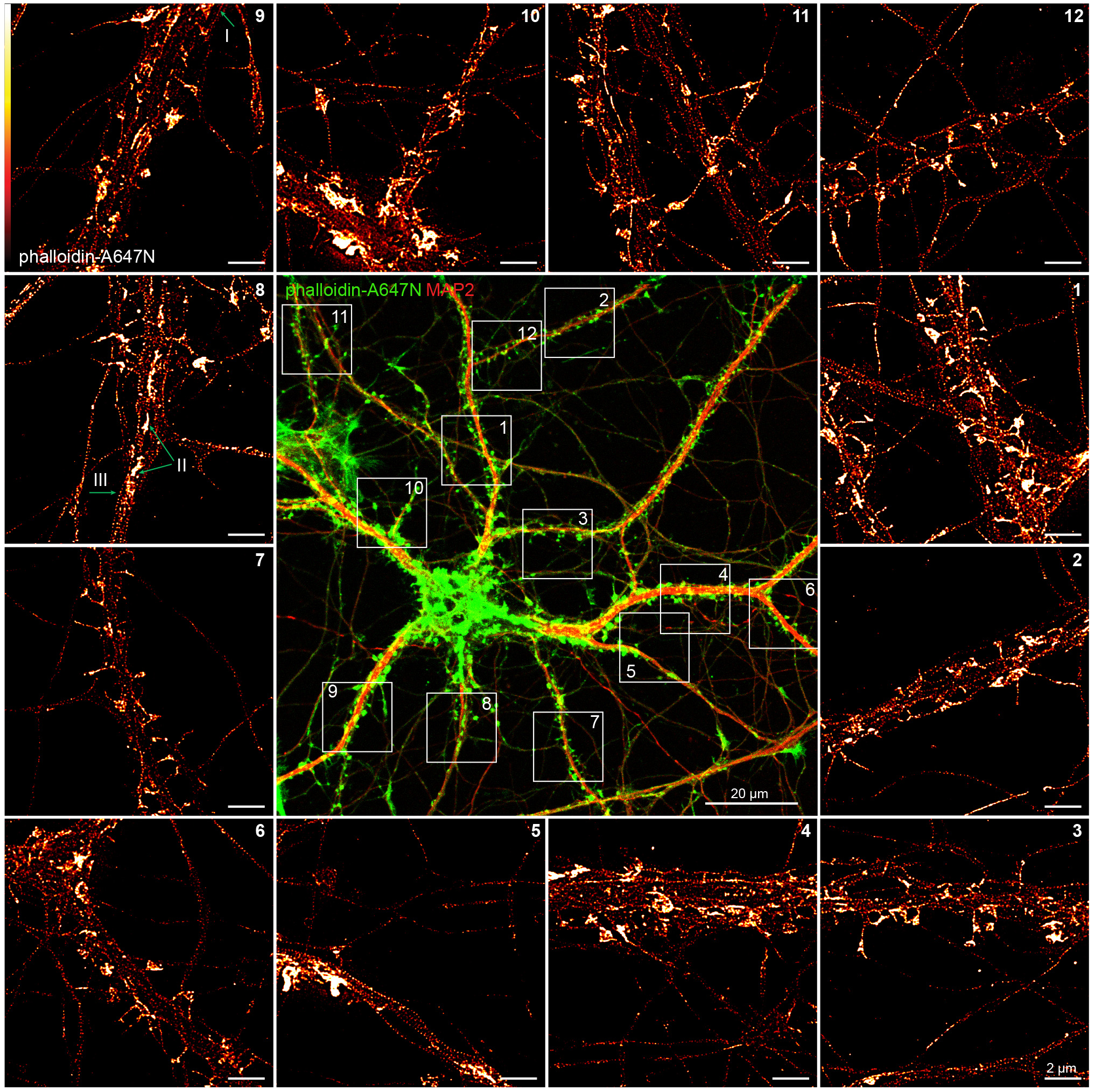
**

**Supplementary Figure 1** Periodic F-actin structures are present in all dendrites in primary hippocampal cultures.

(a) Representative overview confocal image of a primary hippocampal neuron (DIV21) stained with anti-MAP2 (red) antibody and phalloidin-A647N (green) and higher magnifications of deconvolved phalloidin-A647N STED images (pseudo-color) corresponding to the indicated dendrites. Different types of actin filaments in dendrites are indicates by green arrows: I – longitudinal filaments, II – F-actin patches, III – periodic cortical F-actin.

**
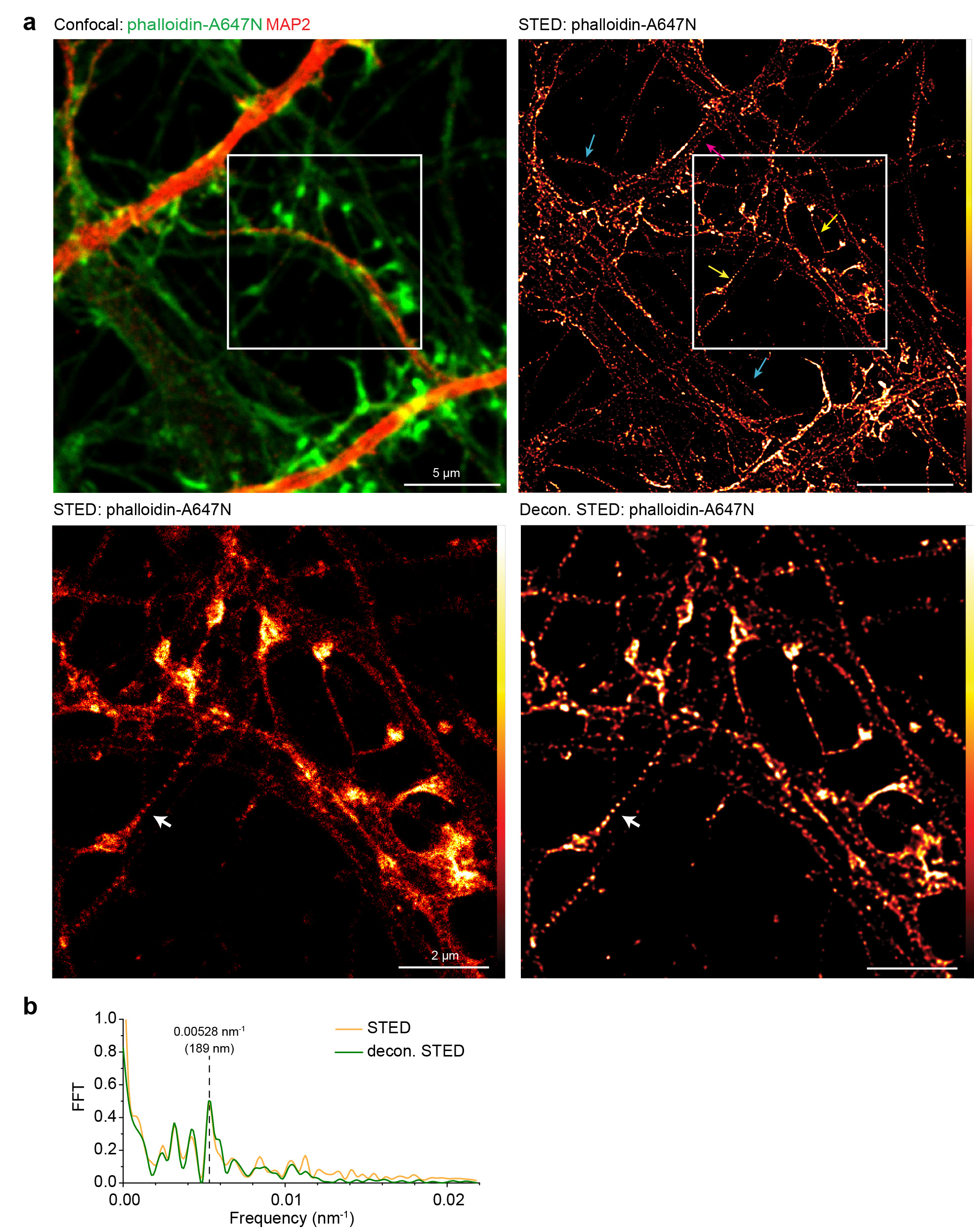
**

**Supplementary Figure 2** Periodic F-actin structures in spines.

(a) Top: Overview confocal and deconvolved STED image of primary hippocampal cultures at DIV21 shows actin rings in axons (blue arrows), dendrites (pink arrow), and in spines (yellow arrows). Bottom: Higher magnification region. Arrow indicates spine used for FFT (b).

(b) Fast Fourier Transformation (FFT) analysis of phalloidin staining of a single spine neck indicated by arrow in (a) shows peak at 189 nm both for raw and deconvolved STED image.


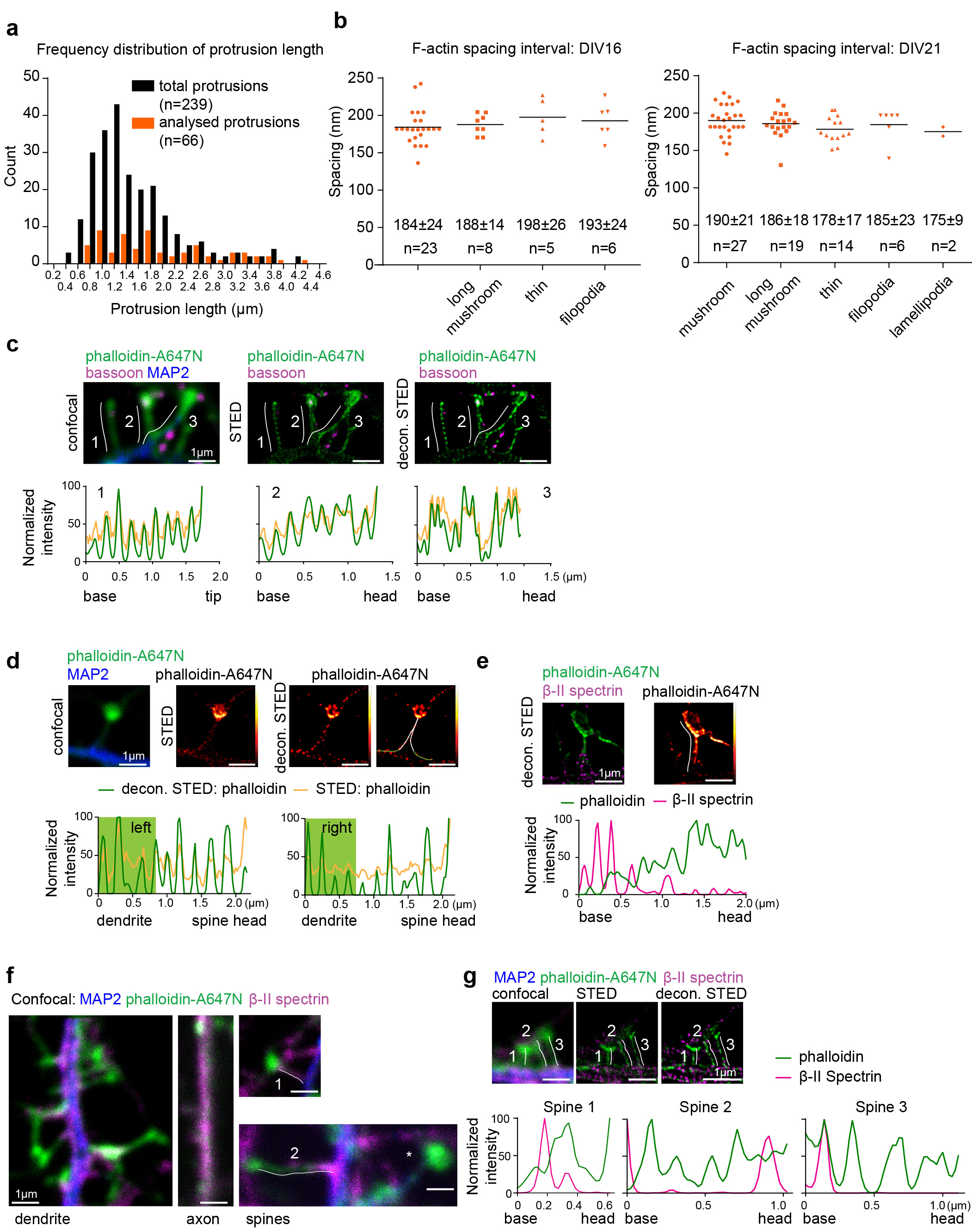


**Supplementary Figure 3** Periodic F-actin structures in different protrusion types.

(a) Frequency distribution of protrusion length for overall spines in DIV21 primary hippocampal cultures (black) and of protrusions used for analysis of F-actin spacing (orange).

(b) Quantification of cortical F-actin periodicity in dendritic spines at DIV16 (left) and DIV21 (right) for different protrusion types does not show differences in F-actin spacing interval. Indicated are mean ± standard deviation (Brown-Forsythe test for equal variances p=0.75, p=0.70; 1-way ANOVA p=0.62, p=0.41, respectively). n numbers are numbers of analyzed protrusions from 1 neuronal preparation, 4 coverslips at DIV16 and 3 independent experiments (neurons from 8 coverslips) at DIV21.

(c) Peridic F-actin structures are present in branched spines. Top: Confocal and corresponding 2-color raw and deconvolved STED images of periodic F-actin in a branched mushroom-like spine (phalloidin-A647N (green) and bassoon (magenta)). Bottom: Normalized intensity profiles of phalloidin staining indicated on top.

(d) Periodic F-actin lattice continuous from the dendrite into spines. Example of confocal and corresponding raw and deconvolved STED images. Normalized intensity profiles along the indicated lines are plotted below. Green color highlights dendritic region.

(e) Periodic F-actin filaments can expand into the spine head. Example of 2-color STED image of phalloidin-A647N (green) and β-II spectrin (magenta, top left) and pseudo-color image of phalloidin-A647N (top right). Normalized intensity profiles in parallel to the indicated line (bottom).

(f) Confocal images of dendrite, axon and different spines in primary cultures, corresponding to Figure 2f, 2g.

(g) Confocal, raw and deconvolved STED images of dendritic spines stained against MAP2 (blue), β-II spectrin (magenta) and phalloidin-Atto647N (green). Normalized intensity profiles of STED images along lines parallel to the indicated ones are shown below. Note that β-II spectrin shows alternating periodic pattern in dendrite but not in the spines.

**
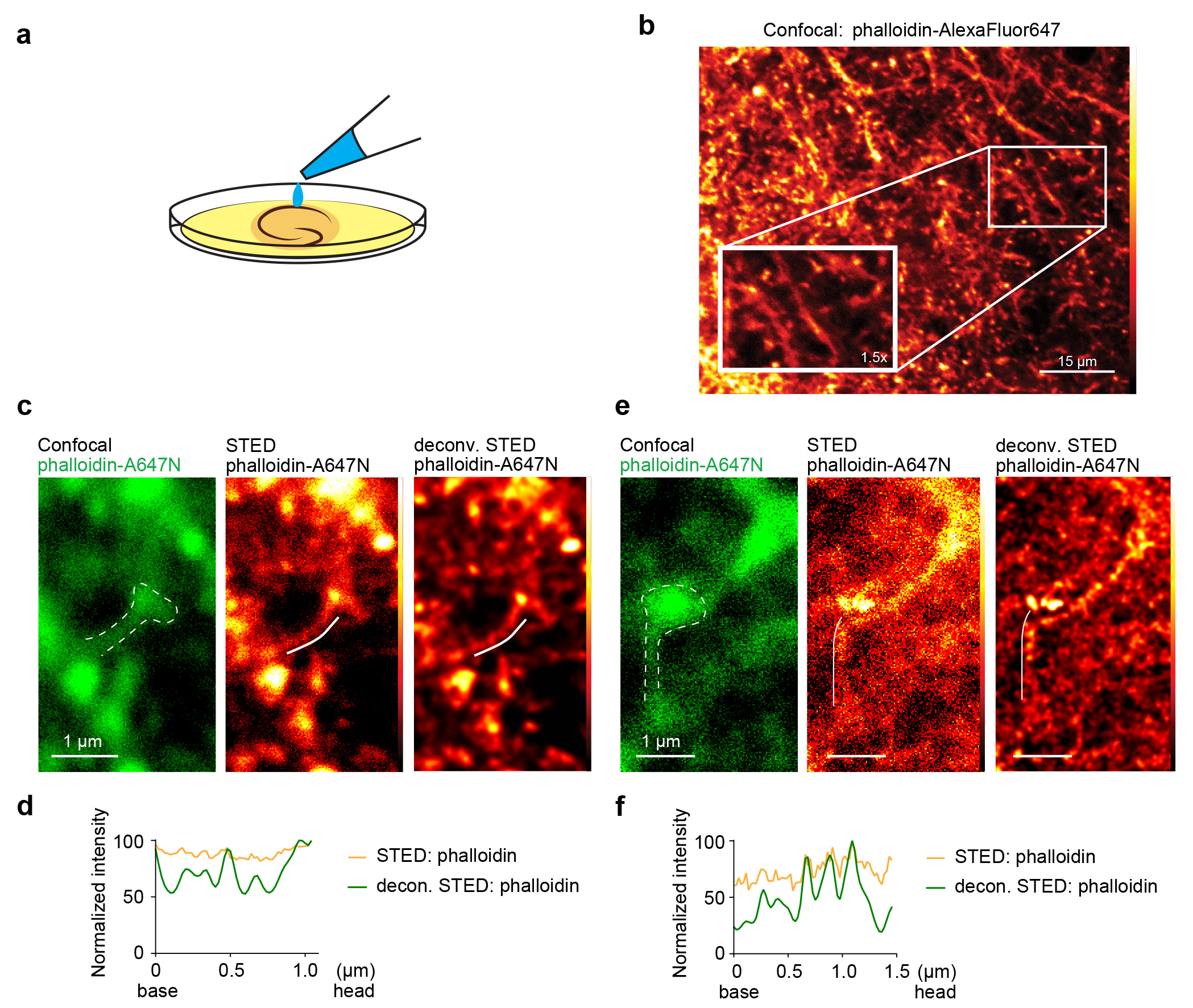
**

**Supplementary Figure 4** Phalloidin-staining in acute and organotypic hippocampal slices.

(a) Cartoon demonstrating the application of phalloidin-Atto647N onto hippocampal slices.

(b) Overview confocal image of an organotypic hippocampal slice stained with phalloidin-AlexaFluor647.

(c) Example STED images of periodic F-actin in dendritic spines in organotypic hippocampal slices visualized by phalloidin-A647N.

(d) Normalized intensity profiles of phalloidin-A647N of the spine indicated in (c).

(e) Representative confocal image of a spine from an acute hippocampal slice with phalloidin-A647N (green, left panel) and corresponding raw and deconvolved STED image (middle and right).

(f) Normalized intensity profiles of phalloidin-A647N of the spine indicated in (e).
